# Association between Short-Term Exposure to Air Pollution and Dyslipidemias among Type 2 Diabetic Patients in Northwest China: A Population-Based Study

**DOI:** 10.3390/ijerph15040631

**Published:** 2018-03-30

**Authors:** Minzhen Wang, Shan Zheng, Yonghong Nie, Jun Weng, Ning Cheng, Xiaobin Hu, Xiaowei Ren, Hongbo Pei, Yana Bai

**Affiliations:** 1 School of Public Health, Lanzhou University, Lanzhou 730000, China; wangmzh@lzu.edu.cn (M.W.); zhengsh@lzu.edu.cn (S.Z.); wengj16@lzu.edu.cn (J.W.); huxiaobin@lzu.edu.cn (X.H.); renxw@lzu.edu.cn (X.R.); peihb@lzu.edu.cn (H.P.); 2 Jinchang Center for Disease Prevention and Control, Jinchang 737100, China; nieyh_cdc@163.com; 3 College of Basic Medicine, Lanzhou University, Lanzhou 730000, China; chengn@lzu.edu.cn

**Keywords:** air pollution, diabetes, total cholesterol, triglycerides, low-density lipoprotein cholesterol, decreased high-density lipoprotein cholesterol

## Abstract

Air pollution exposure may play an adverse role in diabetes. However, little data are available directly evaluating the effects of air pollution exposure in blood lipids of which dysfunction has been linked to diabetes or its complications. We aimed to evaluate the association between air pollution and lipids level among type 2 diabetic patients in Northwest China. We performed a population-based study of 3912 type 2 diabetes patients in an ongoing cohort study in China. Both spline and multiple linear regressions analysis were used to examine the association between short-term exposure to PM_10_, SO_2_, NO_2_ and total cholesterol (TC), triglycerides (TG), low-density lipoprotein cholesterol (LDL-C), and high-density lipoprotein cholesterol (HDL-C). By spline analyses, we observed that the relationship between SO_2_ and HDL-C and LDL-C was shown to be non-linear (*p*_non-lin-association = 0.0162 and 0.000). An inverted U-shaped non-linear relationship between NO_2_ and LDL-C was found (*p*_non-lin-association < 0.0001). A J-shaped non-linear relationship between PM_10_ and TC, HDL-C (*p*_non-lin-association = 0.0173, 0.0367) was also revealed. In linear regression analyses, a 10 μg/m^3^ increment in SO_2_ was associated with 1.31% (95% CI: 0.40–2.12%), 3.52% (95% CI: 1.07–6.03%) and 7.53% (95% CI: 5.98–9.09%) increase in TC, TG and LDL-C, respectively. A 10 μg/m^3^ increment in PM_10_ was associated with 0.45% (95% CI: 0.08–0.82%), 0.29% (95% CI: 0.10–0.49%) and 0.83% (95% CI: 0.21–1.45%) increase in TC, HDL-C and LDL-C, respectively. For NO_2_, an increment of 10 μg/m^3^ was statistically associated with −3.55% (95% CI: −6.40–0.61%) and 39.01% (95% CI: 31.43–47.03%) increase in HDL-C and LDL-C. The adverse effects of air pollutants on lipid levels were greater in female and elder people. Further, we found SO_2_ and NO_2_ played a more evident role in lipid levels in warm season, while PM_10_ appeared stronger in cold season. The findings suggest that exposure to air pollution has adverse effects on lipid levels among type 2 diabetes patients, and vulnerable people may pay more attention on severe air pollution days.

## 1. Introduction

Air pollution has become a global major public health problem. The adverse effects of ambient air pollution on human health, including cardiovascular diseases (CVD) [1], respiratory diseases (RD) [2] and cerebrovascular diseases (CBD) [3], have been established in a series of major epidemiologic and observational studies. Recently, more and more studies have been focused on the potential impacts of exposure to air pollution on increased incidence [4], prevalence [5] or mortality [6] of diabetes mellitus (DM). Moreover, some studies also reported that the associations with air pollution exposure and CVD were stronger among people with DM [7]. Thus, it can be seen that people with DM may be especially vulnerable to exposure to air pollution. 

DM is one of the most common metabolic disorders in the world and the prevalence of diabetes in adults has been increasing in the last decades [8]. According to the International Diabetes Federation (IDF) [9], there were 415 million people with DM aged 20–79 years and three quarters (75%) of those were living in low- and middle-income countries in 2015. As known, high low-density lipoprotein cholesterol (LDL-C), high triglycerides (TG), high total cholesterol (TC), and low high-density lipoprotein cholesterol (HDL-C) are mostly associated with the development of DM and are also well-established risk factors for CVD [10,11,12]. In recent years, some studies found that dyslipidemias, characterized by decreased HDL-C, increased LDL-C, TG and TC, have been associated with elevated air pollutants [13,14,15]. Thus, in a cumulative lifetime exposure to air pollution, the increases of LDL-C, TG, TC and decreases of HDL-C may increase the risk of the development of cardiovascular events, particularly in people with diabetes. 

In China, due to the rapid industrialization, urbanization and increasing energy consumption, air pollution has become a major threat to public health. Numerous studies have illustrated associations between air pollution and various health effects in recent years [16,17,18]. Meanwhile, the prevalence of DM and pre-DM were estimated to be 11.6% and 50.1% in 2013 [19], which indicated that DM has been a most important public health problem in China. However, limited studies have focused on the association between air pollutants and lipid levels among diabetes patients in China, which may be important for air pollution control and DM prevention. 

The Jinchang City, Gansu Province of China is a typical resource mining city. Due to emissions of industrial pollutants and climate arid problems, the air quality in Jinchang is more serious. So, based on the platform of Jinchang cohort study, which has been established in 2011 [20], we aim to investigate the effects of short-term exposure to air pollution (PM_10_, SO_2_ and NO_2_) on the level of TG, TC, HDL-C and LDL-C among type 2 diabetes patients.

## 2. Materials and Methods

### 2.1. Study Area and Population

This study was based on the baseline data collected by the Jinchang cohort study, which was conducted in Jinchang city, Gansu Province, Northwest China. The Jinchang cohort study is an ongoing prospective population-based cohort study. The design and methods of the Jinchang cohort study have been detailed elsewhere [20]. Briefly, the baseline survey was conducted from June 2011 to December 2015 and included 48,001 participants (61.7% men, 38.3% female). In this study, 3912 type 2 diabetic patients were selected as subjects, which was defined as fasting plasma glucose ≥7.0 mmol/L or those who were on anti-diabetic medications at the time of the baseline interview [21]. The study was approved by the Ethical Committees of the Public Health School of Lanzhou University (Ethical Approval Code: 2017-01). 

All participants underwent in-person interviews, comprehensive physical examinations, laboratory tests and biosample collection. The interviews were conducted by trained interviewers using a standardized and structured questionnaire. The information included basic demographic factors (age, sex, education level, work type, and marital status), life style (smoking, alcohol consumption status and physical activity) and other characteristics (e.g., hypertension). The comprehensive physical examination was performed by clinicians, which include a measurement of weight, height, and blood pressure. Automatic recording instruments (SK-X80/TCS-160D-W/H, Sonka, Shenzhen, China) were used to measure weight and height. BMI was calculated as weight in kilograms divided by the square of height in meters. 

Tobacco smoking was categorized as current, former and non-smoker. Current smokers were defined as those who smoked at least one cigarette per day in the last six months. Former smokers were defined as those who used to be smokers, but who had smoked less than one cigarette per day or stopped smoking for at least the past six months. The rest of the participants were defined as non-smoker. Alcohol consumption status was also categorized as current drinkers who drank hard liquor, beer, or wine at least one time per week during the past six months. Former drinkers were those who used to be drinkers, but who had drank less than one time per week or stopped drinking for at least the past six months. Non-drinkers are those who always drank less than once per week or not at all. Hypertension was defined as systolic pressure ≥140 mmHg or diastolic pressure ≥90 mmHg, or self-reported treatment for hypertension.

The biochemical examinations were performed using a clinical chemistry automatic analyzer (Hitachi 7600-020, Kyoto, Japan) during the morning, including TC, TG, HDL-C and LDL-C. The date (year, month and day) of the health examination was recorded for each participant.

### 2.2. Air Pollution and Weather Data

Daily air pollution data were obtained from Jinchang Environmental Monitoring Centre during 2011 to 2015, including particulate matter less than 10 μm in aerodynamic diameter (PM_10_), sulfur dioxide (SO_2_) and nitrogen dioxide (NO_2_). Meanwhile, the weather data per day on mean temperature and relative humidity were collected from Jinchang Bureau of Meteorology during 2011 to 2015. Both air pollution and weather data were matched with the date of health examinations for each subject. 

### 2.3. Statistical Analysis

To consider the lag pattern effect of air pollutants on lipid profiles, we first used spearman’s rand correlation methods to examine the effects with different lag structures, including both single-day lag (from lag0 to lag6) and moving averages of lag01, lag02, lag03, lag04, lag05, lag06 and lag07, respectively. Then, the day with strongest effects was selected to analyze later. 

Next, we used restricted cubic splines functions with 5-knot (5th, 25th, 50th, 75th, 95th percentiles) to examine the associations between PM_10_, SO_2_, NO_2_ and log-transformed TC, TG, HDL-C, LDL-C [22]. The overall associations between three air pollutants and four lipid levels can be revealed by cubic splines. It was also used to find whether there was a non-linear regression relationship. 

Then, multiple linear regression analyses were further conducted. The final multiple linear regression model was adjusted for age, sex, BMI, smoking status (never, current smoker, former smoker), drinking status (never, current drinker, former drinker), education level (no normal education, primary education, middle school, high school, college or higher), work type (management and services, workers), marital status (married, others), hypertension (yes, no) and seasonality (winter (December to February), spring (March to May), summer (June to August) and fall (September to November)). The models were also controlled the non-linear impacts of 3 days’ moving average of mean temperature and relative humidity, using 4 degrees of freedom natural cubic spline [23]. The results are given as percent changes in geometric mean (GM) of TC, TG, HDL-C and LDL-C concentration for 10 μg/m^3^ increment in air pollutant (% change in GM = (10^(10×β)^ – 1) × 100%, where β is the regression coefficient [24].

Finally, we also used the linear regression analyses to check the air pollution effects by sex (female or male), age (<60 years and ≥60 years) and season (cold: October to March; warm: April to September). Statistical analyses were performed using SAS software version 9.2 (SAS Institute Inc., Cary, NC, USA). All reported *p* values were made on the basis of two-side tests with a significance level of 0.05.

## 3. Results

Table 1 provides characteristics of the participants. There were 3912 type 2 diabetic patients in this study, wherein 29.68% were female, 52.22% were ≥60 years old, 46.19% were never smokers, 71.57% were never drinkers, and 49.28% were combined with hypertension. The average level of BMI is 25.26 ± 3.14 kg/m^2^. The median concentration of TC, TG, HDL-C, and LDL-C were 4.80 mmol/L, 1.90 mmol/L, 1.20 mmol/L, and 3.30 mmol/L, respectively.

**Figure 1:**
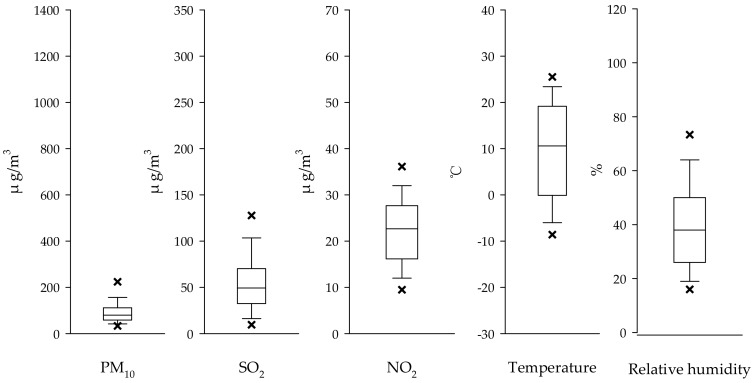
Box plot of PM_10_, SO_2_, NO_2_, Temperature and Relative humidity in Jinchang city, Gansu Province, China, 2011–2015. (× represent the 5th/95th percentiles of each value).

**Figure 2:**
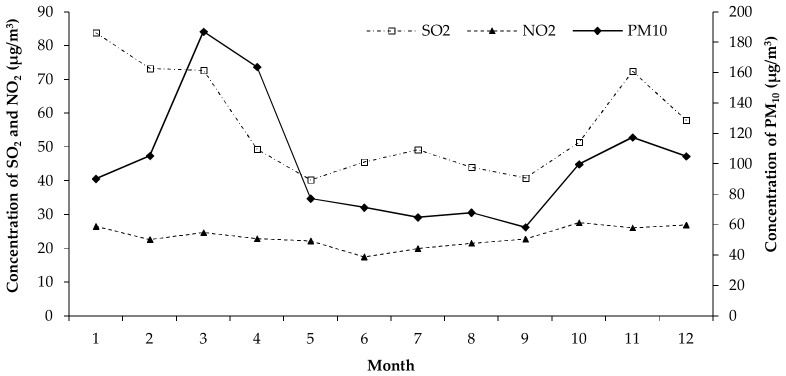
The distribution of PM_10_, SO_2_, NO_2_ in different season in Jinchang city, Gansu Province, China, 2011–2015.

The distribution characteristics of PM_10_, SO_2_, NO_2_, temperature and relative humidity in Jinchang city from 2011 to 2015 were shown in Figure 1. The average daily PM_10_, SO_2_ and NO_2_ concentrations during 2011–2015 were 98.82 μg/m^3^, 54.19 μg/m^3^, and 23.24 μg/m^3^, respectively. In different seasons, the concentration of PM_10_, SO_2_ and NO_2_ was much higher in spring and winter than that in fall and summer (Figure 2). The average daily temperature and relative humidity during 2011–2015 were 11.72 °C and 36.93%.

**Figure 3:**
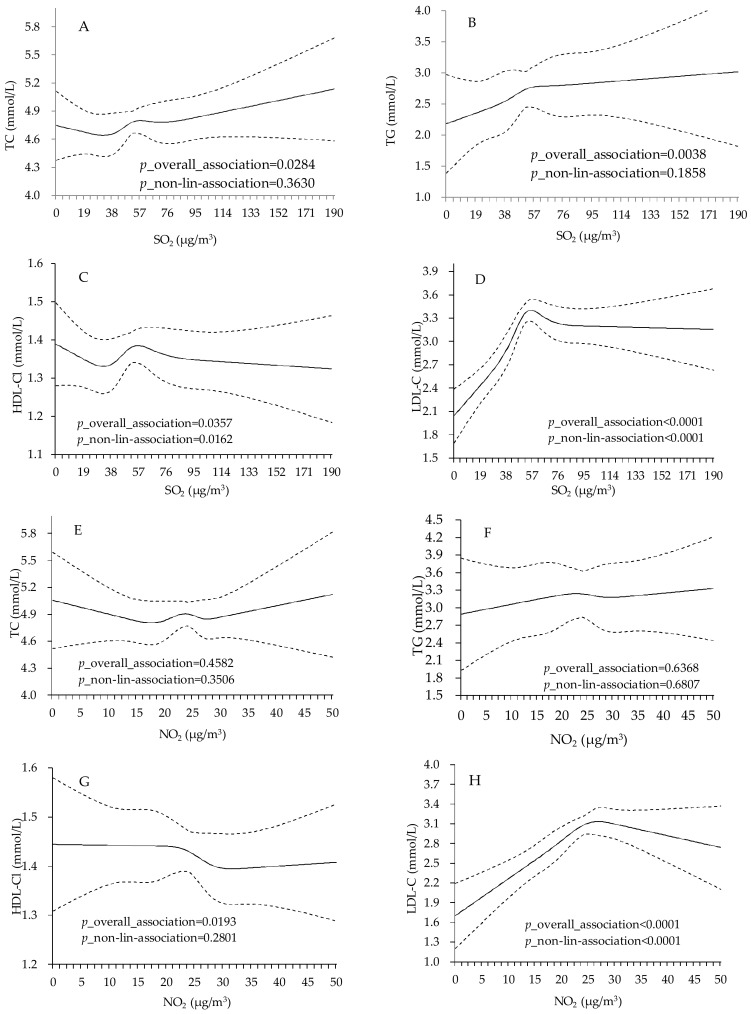

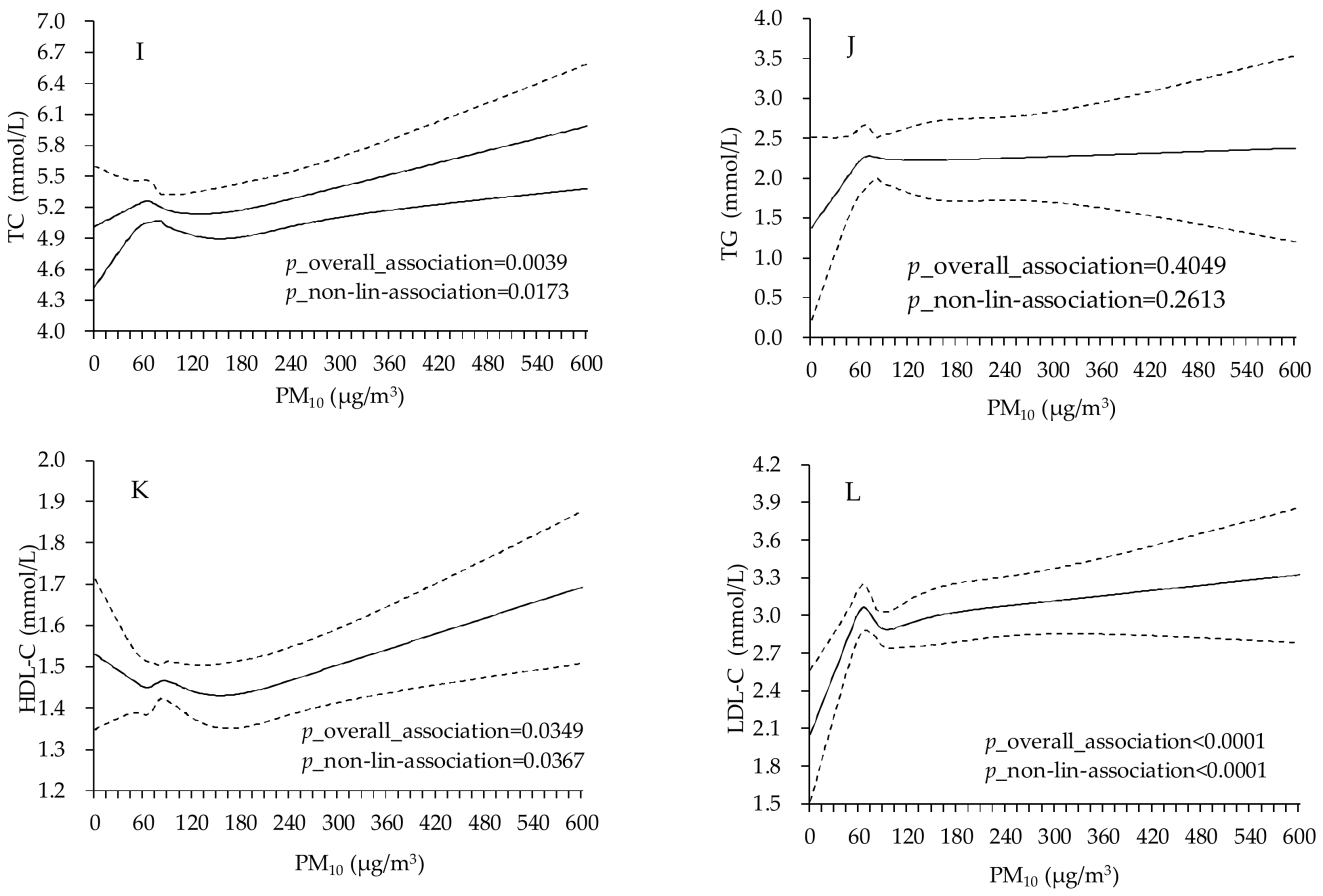
Adjusted dose-response association between PM_10_, SO_2_, NO_2_ and lipid profiles among type 2 diabetes patients in Jinchang, China (*n* = 3912). Adjusted for age, sex, education, smoke status, alcohol drink, occupation, BMI and FPG fitted simultaneously. (**A**–**D**) represent the dose-response association between SO_2_ and TC, TG, HDL-C and LDL-C; (**E**–**H**) represent the dose-response association between NO_2_ and TC, TG, HDL-C and LDL-C; (**I**–**L**) represent the dose-response association between PM_10_ and TC, TG, HDL-C and LDL-C; Solid lines present the predicted values of lipid levels in different concentration of air pollutants. Dashed lines are 95 percent confidence intervals.

After adjusting for covariates, the concentration of SO_2_ was statistically associated with TC, TG, HDL-C and LDL-C (*p*_overall_association = 0.0284, 0.0038, 0.0357, 0.0000) and the relationship between SO_2_ and HDL-C and LDL-C was also shown to be non-linear (*p*_non-lin-association = 0.0162 and 0.000). As with SO_2_, the effects of NO_2_ only shown significant association with HDL-C and LDL-C (*p*_overall_association = 0.0193, 0.000). The relationship between NO_2_ and LDL-C was inverted U-shaped (*p*_non-lin-association < 0.0001). For PM_10_, the effect was statistically associated with TC, HDL-C and LDL-C (*p*_overall_association = 0.0039, 0.0349, 0.0000). We also observed a J-shaped relationship between PM_10_ and TC, HDL-C (*p*_non-lin-association = 0.0173, 0.0367) (Figure 3).

Table 2 showed the linear regression analyses results between air pollutants and log-transferred TC, TG, HDL-C and LDL-C. In model 2, after adjustment demographic, lifestyle, BMI, FPG and hypertension, we observed that 10 μg/m^3^ increment in SO_2_ was associated with a significant increase in TC, TG and LDL-C concentrations, ranging from 1.31 to 7.53% change in GM. A 10 μg/m^3^ increment in PM_10_ was associated with a significant increase in TC, HDL-C and LDL-C concentrations, by 0.45, 0.29 and 0.83% change in GM, respectively. An increment of 10 μg/m^3^ for NO_2_ was statistically associated with −3.55% and 39.01% change in GM increase in HDL-C and LDL-C. 

In addition, we further examined the adverse effects of air pollutants on lipids level among type 2 diabetes patients in different groups (Table 3). Excepted for TG, the effects of PM_10_, SO_2_, and NO_2_ on lipids level were much stronger in females than males. For two age groups, people who aged more than 60 years were much more vulnerable populations than people aged less than 60 years. 

Considering the adverse effect of air pollutants varied in different seasons, we also conducted a stratification analysis by cold and warm season (Table 4). The effect of SO_2_ on TC, TG, HDL-C and LDL-C concentrations among type 2 diabetes patients was statistically much stronger in warm season than cold season, ranging from 1.68 to 24.11% change in GM per 10 μg/m^3^ increment of SO_2_. For NO_2_, the effects on TG and LDL-C were only significant in warm season. An increase of 10 μg/m^3^ of NO_2_ was associated with 10.15% (95% CI: 1.48%, 20.20%) and 69.55% (95% CI: 57.91%, 82.04%) change in GM. However, the effect of PM_10_ on TC, HDL-C and LDL-C concentrations was higher in cold season, ranging from 0.31 to 0.92% change in GM per 10 μg/m^3^ increment of PM_10_.

## 4. Discussion

To our knowledge, this is the first study to examine the effects of air pollutants on lipid levels among type 2 diabetes patients in Northwest China. In this study, we found that short-term exposure to PM_10_, SO_2_ and NO_2_ was significantly associated with blood lipids level among type 2 diabetes patients and a J-shaped or inverted U-shaped relationships was also observed by using spline analyses. In stratification analysis, the effects of air pollutants on lipids levels were stronger on the female and elder population than male and young. In addition, the effects of three air pollutants on lipids level were shown to vary in different seasons. In warm season, the adverse effects of SO_2_ and NO_2_ on TC, TG, HDL-C or LDL-C were much greater, while the effect of PM_10_ on TC, HDL-C and LDL-C was higher in cold season. As we know, dyslipidemia can affect the prognosis of diabetes and contributes significantly to the excess risk of cardiovascular events in diabetes patients [25,26]. These results suggest that air pollution exposure may play a role in development of diabetes and cardiovascular events. 

In the past few years, a number of studies found exposure to air pollutants were significantly associated with increased incidence, mortality and prevalence of type 2 diabetes. Based on 13 studies conducted in Europe or North America, a recent systematic review concluded that there was a positive association of air pollution and type 2 diabetes risk [27]. In China, where air pollution levels are much higher than in developed countries, few epidemiological studies explored the relationship between air pollutants and type 2 diabetes. Ling Tong et al. reported that an increase of 10 μg/m^3^ in a 2-day average concentrations of NO_2_ corresponds to increase in diabetes morbidity of 1.22% (95% CI: 0.51, 2.96) in Tianjin, China [28]. Kan et al. revealed that each increase of 10 mg/m^3^ in PM_10_ or NO_2_ was found to correspond to a 1.006 (95% CI: 1.000–1.012) or 1.013 (95% CI: 1.000–1.026) relative risk of diabetes mortality in Shanghai [29]. Brook et al. indicated fine particulate matter (PM_2.5_) were significantly associated with worsening insulin resistance (0.22 (95% CI: 0.08–0.36) unit increase per SD increase of ambient fine particulate matter on lag days 5) in Beijing [30]. 

Among diabetes mellitus patients, the lipid abnormalities are more prevalent. Dyslipidemia is one of the major risk factors for cardiovascular disease in hyperglycemic patients, which leads to death among diabetes patients. The result of this study might be used to interpret the underlying biological mechanism of the association between air pollution and diabetes mortality or morbidity. Interestingly, we found that the impacts of PM_10_, SO_2_ and NO_2_ showed some adverse effect on blood lipid level among type 2 diabetes patients. Moreover, four lipids have different sensitivity to three air pollutants. Some previous epidemiology and animal toxicology studies were consistent with our results. Based on 11,623 adult participants of NHANES III, a study showed an interquartile range (11.1 μg/m^3^) increase in PM_10_ was associated with 1.43% greater total cholesterol (95% CI: 1.21–1.66), 2.42% greater serum triglycerides (95% CI: 1.09, 3.76), and 1.18% greater LDL-C (95% CI: 0.81, 1.56), but the effect on HDL-C was modest [15]. Cai et al. focused on 144,082 participants and found an IQR higher PM_10_ (2.0 µg/m^3^) or NO_2_ (7.4 µg/m^3^) was associated with higher triglycerides 1.9% (95% CI: 1.5–2.4%) and 2.2% (95% CI: 1.6–2.7%); however, only NO_2_ showed an adverse effect on HDL-C [0.5% (95% CI: 0.3–0.8) per 7.4 μg/m^3^ increased of NO_2_] [31]. Among 1413 students aged 14–18 years, a study used multiple linear regressions model and found linear regressions based on correlation of coefficients of the air quality index (AQI) with cardio metabolic risk factors showed significant positive correlations of AQI with total cholesterol, LDL-cholesterol, and triglycerides, as well as significant negative correlations with HDL-cholesterol [32]. Marie-Abele et al. showed among participants with LDL cholesterol <80 mg/dL, that an IQR increase in PM_2.5_ exposure was associated with a 7 mg/dL (95% CI: 5, 10) increase in LDL cholesterol, whereas among subjects with LDL cholesterol levels close to 160 mg/dL, the same exposure was related to a 16 mg/dL (95% CI: 13, 20) increase in LDL cholesterol [33]. An animal toxicology experiment revealed that sub-chronic exposure to SO_2_ resulted in a significant dose-dependent increasing in plasma triglycerides, up to +363% in the 10 ppm cholesterol enriched exposed animals [34]. 

The effect of air pollutants on lipid level among type 2 diabetes patients was much stronger in female and people aged ≥60 years than male and people aged <60 years. The results were consistent with some previous studies [23]. In addition, some studies, which reported the air pollutions effect on the morbidity or mortality risks of diabetes, also supported our results [28,35]. This indicated the women and elderly are more sensitive than men and younger people to the air pollution because of physiology. Also, the prevalence of cardiovascular and respiratory diseases in older people is higher, which may lead to elderly more vulnerable to air pollution. 

In this paper, we further found that the effects of air pollution on lipids level varied in different seasons. Although the concentrations of SO_2_ and NO_2_ were higher in winter and spring, their effects on TC, TG, HDL-C or LDL-C among type 2 diabetes patients were much stronger in warm season. However, the adverse effect of PM_10_ on lipids level showed a different seasonal effect from SO_2_ and NO_2_. It was observed that PM_10_ exposure played a much stronger role in the increment of TC, HDL-C and LDL-C concentrations among type 2 diabetes patients in cold season. So far, the evidence was not consistent about the modification effects of season on air pollution. Robert et al. reported that NO_2_ and PM_10_ exposure showed much higher adverse effects on hospitalization for diabetes acute complications in cold season, while the effect of SO_2_ was much greater in warm season [36]. Ae et al. found that both of PM_10_, SO_2_ and NO_2_ exposure had a much stronger effect on relative risk of non-accidental, cardiovascular and respiratory mortality in Seoul, Korea in warm season than those in cold season [37]. However, Kan et al. revealed the effects of PM_10_, SO_2_ and NO_2_ on the relative risk of mortality in Shanghai, China were more evident in cool season than in the warm season [38]. In general, the health effects of air pollution may show a variety of characteristic in different countries, as different climate conditions and pollutant sources. 

This is the first study to investigate the association between exposure to air pollution and lipid profiles among type 2 diabetes patients in Northwest China. Based on the baseline data of Jinchang Cohort, potential confounders were controlled, such as sex, age, BMI, smoking, drinking, etc. Although there was some strength in the study, there were also some limitations. First, we used the data of outdoor air pollution monitoring to estimate individual rather than exposures than personal air pollution exposure data. Meanwhile, we did not consider the indoor air pollution and time spent outdoors. This may result in the effects of air pollutants on lipid profiles among type 2 diabetes patients were underestimated. Second, we did not account for a dietary pattern that may be associated with lipid concentration [39]. In our future research, we will focus on the improvement of the exposure data and more potential confounders considering. 

## 5. Conclusions

In summary, exposure to air pollutants (PM_10_, SO_2_, and NO_2_) was associated with an increased level of lipid profiles among type 2 diabetes patients. Given the strong evidence that dyslipidemia among type 2 diabetes patients is an important independent predictor for cardiovascular diseases (CVD) [40], our findings might implicate the enhanced risk of CVD among type 2 diabetes in severe air pollution areas. In addition, we also found female and elderly people were more sensitive to air pollution effects. Thirdly, the modification effect of season on air pollution revealed that gaseous pollutants, including SO_2_ and NO_2_, played more evident role in lipids level in warm season, while particulate matter pollutant, such as PM_10_, showed stronger effects in cold season. Our results should promote further studies on the effect of air pollution on diabetes and support governments to reduce the high-level of air pollution in China.

## Figures and Tables

**Table 1:** Baseline characteristics of the 3912 type 2 diabetes patients.

Variable	Total	Male	Female
N (*n*, %)	3912 (100)	2751 (70.32)	1161 (29.68)
Age (*n*, %)			
<60 years	1869 (47.78)	1368 (49.73)	501 (43.15)
≥60 years	2043 (52.22)	1383 (50.27)	660 (56.85)
Education (*n*, %)			
No normal education	1192 (30.47)	782 (28.43)	410 (35.31)
Primary education	1225 (31.31)	834 (30.32)	391 (33.68)
Middle school	866 (22.14)	610 (22.17)	256 (22.05)
High school	380 (9.71)	314 (11.41)	66 (5.68)
College or higher	249 (6.37)	211 (7.67)	38 (3.24)
Work type (*n*, %)			
Management and services	1166 (29.81)	741 (26.94)	425 (36.61)
workers	2746 (70.19)	2010 (73.06)	736 (63.39)
Married (*n*, %)	3387 (86.58)	2484 (90.29)	903 (77.78)
Smoking (*n*, %)			
Never	1807 (46.19)	697 (25.34)	1110 (95.61)
Current smoker	1400 (35.79)	1364 (49.58)	36 (3.10)
Former smoker	705 (18.02)	690 (25.08)	15 (1.29)
Drinking (*n*, %)			
Never	2800 (71.57)	1662 (60.41)	1138 (98.02)
Current drinker	701 (17.92)	682 (24.79)	19 (1.64)
Former drinker	411 (10.51)	407 (14.79)	4 (0.34)
Hypertension (*n*, %)	1928 (49.28)	1320 (47.98)	608 (52.37)
BMI (kg/m^2^) (mean, SD)	25.26 (3.14)	25.14 (2.94)	25.54 (3.53)
Total cholesterol (TC) (mmol/L) (median, 25th, 75th)	4.80 (4.20, 5.50)	4.70 (4.10, 5.40)	5.00 (4.40, 5.80)
Triglycerides (TG) (mmol/L) (median, 25th, 75th)	1.90 (1.40, 2.90)	1.97 (1.40, 3.00)	1.80 (1.30, 2.60)
High-density lipoprotein cholesterol (HDL-C) (mmol/L) (median, 25th, 75th)	1.20 (1.02, 1.41)	1.16 (0.99, 1.37)	1.29 (1.11, 1.52)
Low-density lipoprotein cholesterol (LDL-C) (mmol/L) (median, 25th, 75th)	3.03 (2.43, 3.61)	3.04 (2.49, 3.59)	3.01 (2.26, 3.65)
FPG (mmol/L) (median, 25th, 75th)	8.00 (7.10, 9.80)	8.00 (7.20, 9.90)	8.00 (7.10, 9.70)

**Table 2:** Percent change in geometric mean (GM) of TC, TG, HDL-C and LDL-C per 10 μg/m^3^ increase in air pollutants among type 2 diabetes patients.

Air Pollutants	Models	TC	TG	HDL-C	LDL-C
		% Change in GM (95% CI)	% Change in GM (95% CI)	% Change in GM (95% CI)	% Change in GM (95% CI)
SO_2_	Model1 ^a^	1.21 (0.38, 2.04) *	3.97 (1.58, 6.41) *	0.13 (−0.70, 0.98)	8.78 (7.26, 10.33) *
	Model2 ^b^	1.31 (0.40, 2.12) *	3.52 (1.07, 6.03) *	0.59 (−0.31, 1.51)	7.53 (5.98, 9.09) *
PM_10_	Model1 ^a^	0.49 (0.13, 0.85) *	0.03 (−0.94, 1.01)	0.31 (0.12, 0.51) *	0.65 (0.05, 1.26) *
	Model2 ^b^	0.45 (0.08, 0.82) *	−0.01 (−1.01, 1.00)	0.29 (0.10, 0.49) *	0.83 (0.21, 1.45) *
NO_2_	Model1 ^a^	1.34 (−0.86, 3.60)	6.28 (0.08, 12.87) *	−2.91 (−5.46, −0.30) *	43.63 (36.02, 51.67) *
	Model2 ^b^	1.16 (−1.06, 3.43)	5.58 (−0.62, 12.16)	−3.55 (−6.40, −0.61) *	39.01 (31.43, 47.03) *

^a^ Adjusted for age, sex, BMI, education, smoke status, alcohol drink, occupation, FPG and hypertension; ^b^ Additionally adjusted for PM_10_, SO_2_ and NO_2_ fitted simultaneously; * *p* < 0.05.

**Table 3:** Percent change in GM of TC, TG, HDL-C and LDL-C per 10 μg/m^3^ increase in air pollutants among type 2 diabetes patients in different groups.

Groups	Air Pollutants	TC	TG	HDL-C	LDL-C
		% Change in GM (95% CI)	% Change in GM (95% CI)	% Change in GM (95% CI)	% Change in GM (95% CI)
Gender					
Male	SO_2_	1.15 (0.19, 2.13) *	4.39 (1.50, 7.36) *	−0.26 (−1.25, 0.74)	7.49 (5.81, 9.20) *
	PM_10_	0.59 (0.09, 1.09) *	−0.18 (−1.57, 1.23)	0.28 (0.00, 0.57) *	0.47 (−0.33, 1.27)
	NO_2_	1.90 (−0.86, 4.74)	5.77 (−2.12, 14.30)	−2.72 (−5.81, 0.46)	41.40 (32.91, 50.44) *
Female	SO_2_	1.79 (0.05, 3.55) *	2.67 (−1.79, 7.34)	1.17 (−0.51, 2.87)	15.34 (11.78, 19.02) *
	PM_10_	0.37 (−0.17, 0.91)	0.12 (−1.21, 1.46)	0.34 (0.08, 0.60) *	0.79 (−0.18, 1.78)
	NO_2_	0.85 (−2.92, 4.77)	9.19 (−0.64, 20.01)	−3.67 (−8.19, 1.08)	49.07 (33.02, 67.06) *
Age					
<60	SO_2_	0.47 (−0.62, 1.58)	1.53 (−1.83, 5.00)	−0.81 (−1.95, 0.34)	2.22 (0.34, 4.14)*
	PM_10_	0.14 (−0.45, 0.74)	−0.87 (−2.59, 0.88)	0.14 (−0.21, 0.49)	0.56 (−0.41, 1.55)
	NO_2_	1.26 (−1.71, 4.31)	1.65 (−6.91, 11.00)	−2.95 (−6.57, 0.81)	9.02 (1.23, 17.40) *
≥60	SO_2_	1.88 (0.51, 3.26) *	6.96 (3.24, 10.81) *	1.69 (0.35, 3.05) *	20.51 (17.84, 23.24) *
	PM_10_	0.53 (0.03, 1.02) *	1.04 (−0.22, 2.32)	0.33 (0.10, 0.56) *	1.13 (0.25, 2.01) *
	NO_2_	2.39 (−0.99, 5.89)	9.31 (0.34, 19.09) *	−0.79 (−4.55, 3.12)	109.23 (92.57, 127.32) *

* *p* < 0.05.

**Table 4:** Percent change in GM of TC, TG, HDL-C and LDL-C per 10 μg/m^3^ increase in air pollutants among type 2 diabetes patients in different seasons.

Groups	Air Pollutants	TC	TG	HDL-C	LDL-C
		% Change in GM (95% CI)	% Change in GM (95% CI)	% Change in GM (95% CI)	% Change in GM (95% CI)
warm	SO_2_	1.68 (0.37, 3.00) *	7.93 (4.04, 11.96) *	1.88 (0.46, 3.32) *	24.11 (21.43, 26.85) *
	PM_10_	0.05 (−0.16, 0.26)	0.54 (−1.00, 2.10)	0.23 (−0.02, 0.48)	0.16 (−0.83, 1.15)
	NO_2_	2.62 (−0.43, 5.76)	10.45 (1.481, 20.20) *	−2.05 (−5.61, 1.63)	69.55 (57.91, 82.04) *
cold	SO_2_	0.84 (−0.34, 2.03)	1.58 (−1.70, 4.96)	−0.68 (−1.75, 0.40)	−0.86 (−2.64, 0.95)
	PM_10_	0.31 (0.04, 0.58) *	0.01 (−1.33, 1.36)	0.34 (0.04, 0.65) *	0.92 (0.17, 1.67) *
	NO_2_	0.47 (−3.08, 4.16)	2.22 (−6.98, 12.33)	−2.77 (−6.55, 1.17)	7.86 (−2.42, 19.22)

* *p* < 0.05.

## Abbreviations

DM	Diabetes mellitus
CVD	Cardiovascular diseases
TC	Total cholesterol
TG	Triglycerides
LDL-C	Low-density lipoprotein cholesterol
HDL-C	High-density lipoprotein cholesterol
FPG	Fasting plasma glucose
BMI	Body mass index
GM	Geometric mean
